# Congenital Limb Deficiency: A Case Report

**DOI:** 10.31729/jnma.7486

**Published:** 2022-05-31

**Authors:** Surendra Khanal, Uttam Pachya, Sushma Thapaliya, Sagar Rana Magar, Bishal Panthi, Arun Khatri

**Affiliations:** 1 Tribhuwan University, Teaching Hospital, Maharajgunj, Kathmandu, Nepal; 2 Gulmi Hospital, Tamghas, Gulmi, Nepal; 3 Hospital for Advanced Medicine and Surgery, Dhumbarahi, Kathmandu, Nepal; 4 Patan Academy of Health Sciences, Lagankhel, Patan, Nepal

**Keywords:** *amelia*, *antenatal care*, *congenital limb deformities*, *fetal ultrasonography*

## Abstract

The complete absence of limbs is a rare occurrence. Though the causes are various, it is hard to elicit most of the time. They are usually diagnosed via anomaly scan but the lack of access to the same can often lead to a term presentation. It is still not uncommon to receive pregnant patients at term to the hospital or in labour as the first antenatal visit. Increasing the feasibility of the scan can help in the early diagnosis and management. Here, we report a rare combination of limb defects that we managed in a district-level hospital and highlight the difficulties in the management and referral of the patients while working in rural areas.

## INTRODUCTION

The majority of congenital limb deficiencies are sporadic with no risk of recurrence while some are associated with syndromes.^1^ The etiologies of congenital limb deficiencies have been classified as genetic, chromosomal abnormalities, environmental, or prenatal diagnostic procedures.^2^ Limb defects can present as an isolated case or with other anomalies like body wall defects or syndromes. The incidence of the complete absence of one or more limbs is 1.41 per 100,000.^3^ Given the rarity of the incidence of the case, we have reported a case of congenital limb deficiency that presented in a rural district hospital.

## CASE REPORT

**Figure 1:**
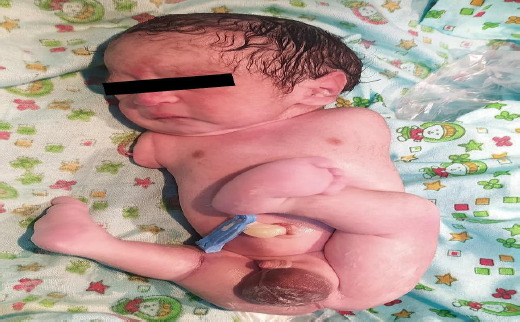
Complete absence of the left upper limb bud and club foot along with a fusion of three lateral digits on the left lower limb.

**Figure 2:**
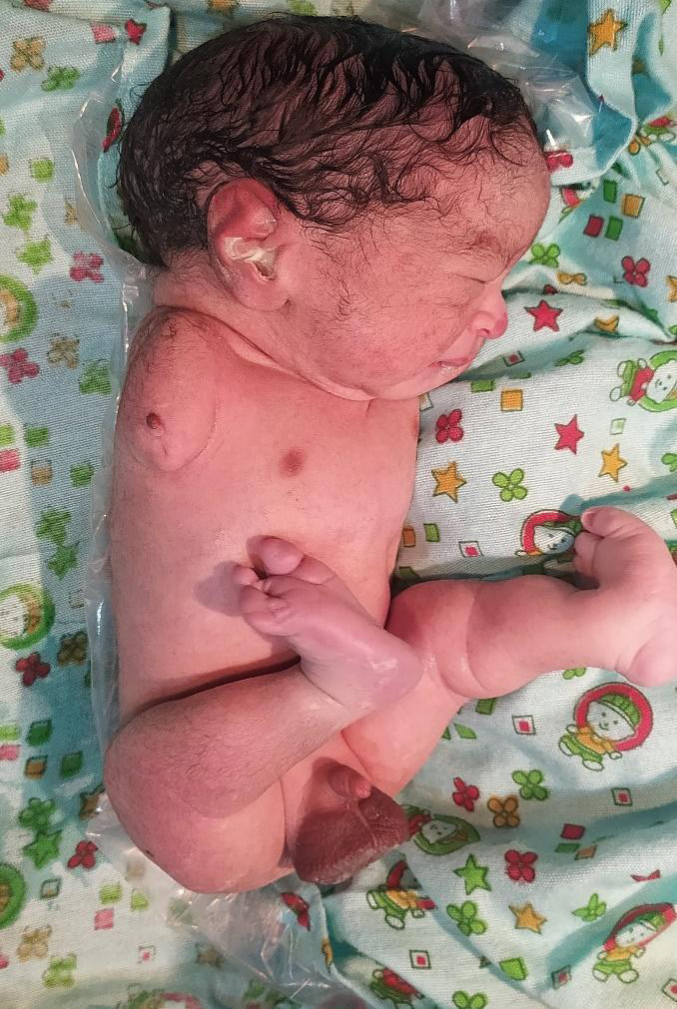
Absence of the right upper limb along with a limb bud. There is also a fusion of the lateral three digits in the right foot.

A 20-year-old primigravida presented to our centre in labour pain. Ultrasound screening revealed a fetus in breech presentation. The baby was estimated to be at 38+^3^ weeks of gestation as per the calculation based on the ultrasonography done in the first trimester. The mother had a total of 2 Antenatal Care (ANC) visits, skipped an anomaly scan, and had not taken folic acid. She did not recall any history of exposure to drugs or specific teratogens during pregnancy. The mother had been healthy throughout her life and was a nonsmoker and didn’t drink alcohol. She had no history of any infections during the pregnancy. There was no family history of congenital birth defects. There was no history of consanguinity. The father had three children, all born normally and living healthy from his previous marriage. The parents were counseled about the risks of breech delivery and methods of delivery. A male baby was born at 38+^3^ weeks of gestation by emergency lower segment cesarean section. The baby had an Appearance, Pulse, Grimace, Activity, and Respiration (APGAR) score of 6/10 and 8/10 at 1 and 5 minutes of birth. On examination, the baby had a right upper limb bud but there was no bud in the left upper limb. The 3 lateral digits were fused in the bilateral foot and club foot deformity was present on the left side (Figure 1, 2).

On examination, there was tenderness in the movement of the right hip. A head and neck examination revealed no obvious abnormalities. There was equal air entry in bilateral lungs and the heart sounds were normal. The abdomen was soft and non-distended, and external genitalia showed well-formed scrotal rugae and bilaterally descended testes. The baby breast-fed normally after birth and passed urine and stool within the first 24 hours of birth.

**Figure 3:**
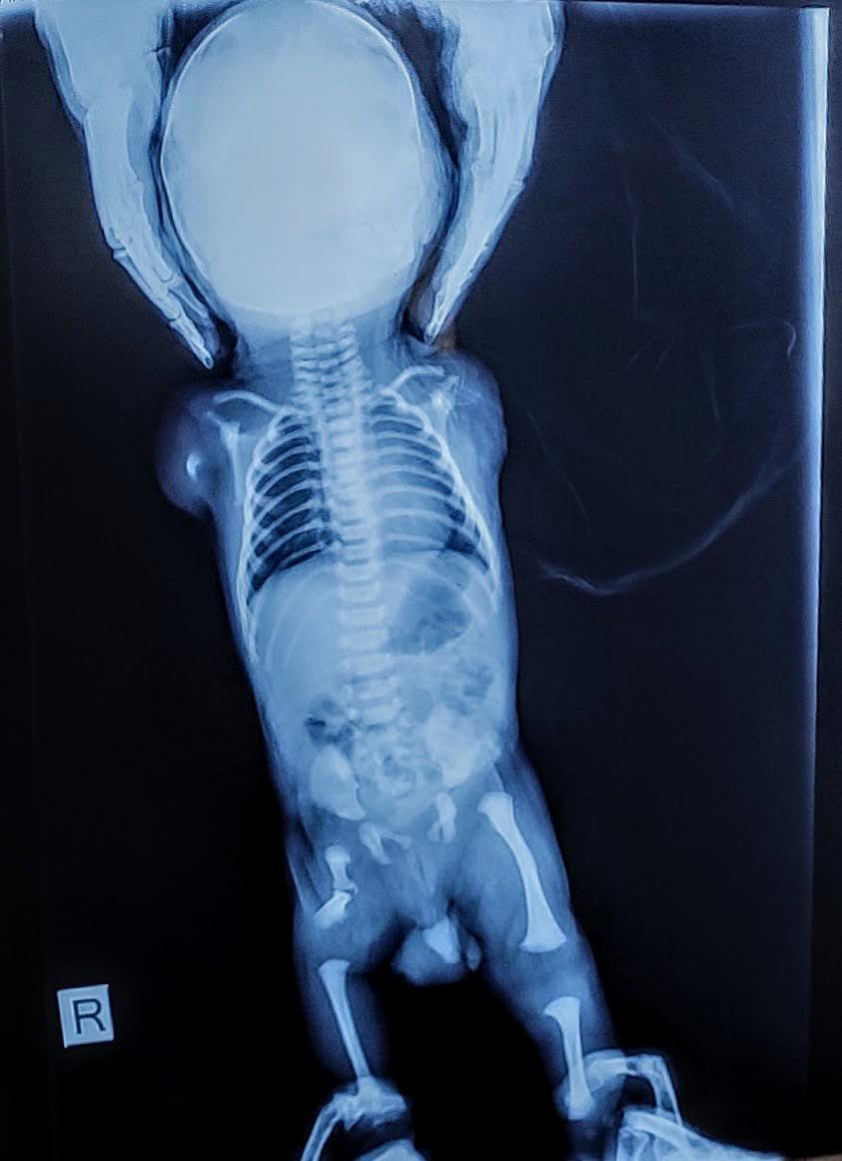
The X-ray of the baby shows a tiny bud on the right upper limb and hypoplasia of the right femur

A complete X-ray of the baby was done after birth and showed a complete absence of the left upper limb and the presence of a limb bud in the right. The right femur was hypoplastic and club foot deformity was present in the left foot. Apart from it, the lungs and heart of the child appeared normal. Complete blood count and renal function tests were done immediately after birth which were reported normal. The parents were counselled regarding the prognosis of the condition and for the early management of clubfoot deformity. The immediate referral was not possible for financial difficulties and the patient had to be referred only on the 3^rd^ day of birth (Figure 3).

## DISCUSSION

Though limb defects are some of the most common birth defects, amelia, the complete absence of limbs is a rare congenital finding.^4^ The combination of limb defects in isolated limb deficiencies shows individual variations. The combination we encountered, in this case, is unique and to our best knowledge, the individual combination of all these defects might be the rarest of the rare.

Limb buds start developing in the ventrolateral body wall around the 26^th^ day of gestation.^5^ Various insults during the developmental period can lead to congenital malformations. A population-based case-control study from Finland showed the association between pregestational diabetes, male sex, and primiparity.^6^ The aetiology of limb deficiencies is difficult to identify. The asymmetry in the findings of limb defects in our case puts amniotic band syndrome as a potential cause.^7^ However, chromosomal abnormalities or various syndromes could also be the cause. A study in an attempt to classify the causes showed that, in more than a third of the cases, the cause was not immediately clear and it was not possible to identify even after autopsy examination.^2^

Our case reflects the importance of antenatal ultrasound scans in the diagnosis of congenital malformations. The routine anomaly scan is best done in the second trimester between the 18^th^ and 22^nd^ weeks.^8^ Scans in the first trimester are routinely done for dating and maternal complaints of vaginal bleedings. However, trans-abdominal and transvaginal scans may also be used to diagnose congenital limb defects even in the first trimester.^9^ In the context of Nepal, women in rural areas are less likely to receive proper antenatal care compared to women in urban areas.^10^

The case was managed in a district-level hospital with no advanced facilities. The lack of genetic studies and neonatologists limited our study. Similarly, the lack of orthopaedic surgeon facilities in our centre limited the management of the case for which the patient was referred to a higher centre.

## CONCLUSIONS

This case highlights the importance of antenatal checkups and anomaly scans in the early diagnosis and counseling of congenital anomalies. Making antenatal checkups and anomaly scans feasible in rural areas can help identify birth defects earlier and help in the management.
